# Spontaneous Rupture of an Ovarian Artery Aneurysm in the Early Postpartum Period: A Case Report

**DOI:** 10.7759/cureus.65137

**Published:** 2024-07-22

**Authors:** Luke Speier, Trevor Ward, Jeffrey Bednar, Nicholas Kramer, Leanne Almario

**Affiliations:** 1 College of Osteopathic Medicine, Touro University Nevada, Henderson, USA; 2 Obstetrics and Gynecology, Red Rock Obstetrics and Gynecology, Las Vegas, USA

**Keywords:** critical care in obstetrics, interventional radiology guided embolization, massive blood loss, post partum hemorrhage, ovarian artery aneurysm

## Abstract

The spontaneous rupture of an ovarian artery aneurysm (OAA) is an extremely uncommon and life-threatening event. Here, we describe the case of a 34-year-old G6P5015 female who underwent spontaneous vaginal delivery. Following delivery, she experienced hypotension and reported right-sided abdominal pain. A contrast-enhanced computed tomography (CT) angiogram revealed an aneurysmal dilation, extravasation, pseudoaneurysms, and a large retroperitoneal hematoma attributable to a rupture of the right ovarian artery. Subsequently, an exploratory laparotomy was performed, and then a transcatheter arterial embolization (TAE) by interventional radiology (IR). At a proximal site, IR successfully embolized both the ovarian and uterine arteries. This case highlights the significance of rapid intervention in managing an OAA. Additionally, we discuss the risk factors and treatment alternatives for OAA, underscoring the importance of considering it in the differential diagnosis when encountering atypical hypotension in the postpartum period.

## Introduction

A spontaneous rupture of an ovarian artery aneurysm (OAA) is an incredibly rare and typically life-threatening event that is associated with pregnancy [1]. Ovarian artery rupture has a hyperacute course, often with rapid clinical deterioration. Early diagnosis is crucial to patient survival, but the rarity of such cases presents a challenge to timely diagnosis. Pregnancy-related changes contribute to over half of ruptured aneurysms in women under 40 years of age. The hemodynamic and endocrine changes that occur during pregnancy likely cause the formation of new aneurysms and the growth of pre-existing aneurysms [2]. These physiologic changes in pregnancy are likely due to the increased pressure of the gravid uterus on the aorta, which causes structural changes in the arteries. Furthermore, increased utero-ovarian artery perfusion may increase aneurysm formation [3]. In the postpartum period, it is normal for branches of the ovarian artery to involute, though failure to do so completely may lead to aneurysms or pseudoaneurysms in subsequent pregnancies [4]. The literature suggests that multiparity is a risk factor for ovarian artery aneurysms and pseudoaneurysms [3,5]. Other risk factors for ovarian aneurysms include uterine fibroids, hypertension, and vigorous uterine massage [6,7]. 

In this report, we describe a case involving a 34-year-old woman who experienced a spontaneous rupture of her right ovarian artery following a spontaneous vaginal delivery, resulting in retroperitoneal hemorrhage and hematoma. The diagnosis was confirmed through a CT angiogram of the abdomen and pelvis after the patient presented with a sudden drop in blood pressure and right-sided flank pain. Subsequent management involved an emergency exploratory laparotomy to ligate the bilateral ovarian arteries, followed by transcatheter arterial embolization (TAE) to occlude the bilateral ovarian and uterine arteries.

## Case presentation

**Figure 1:**
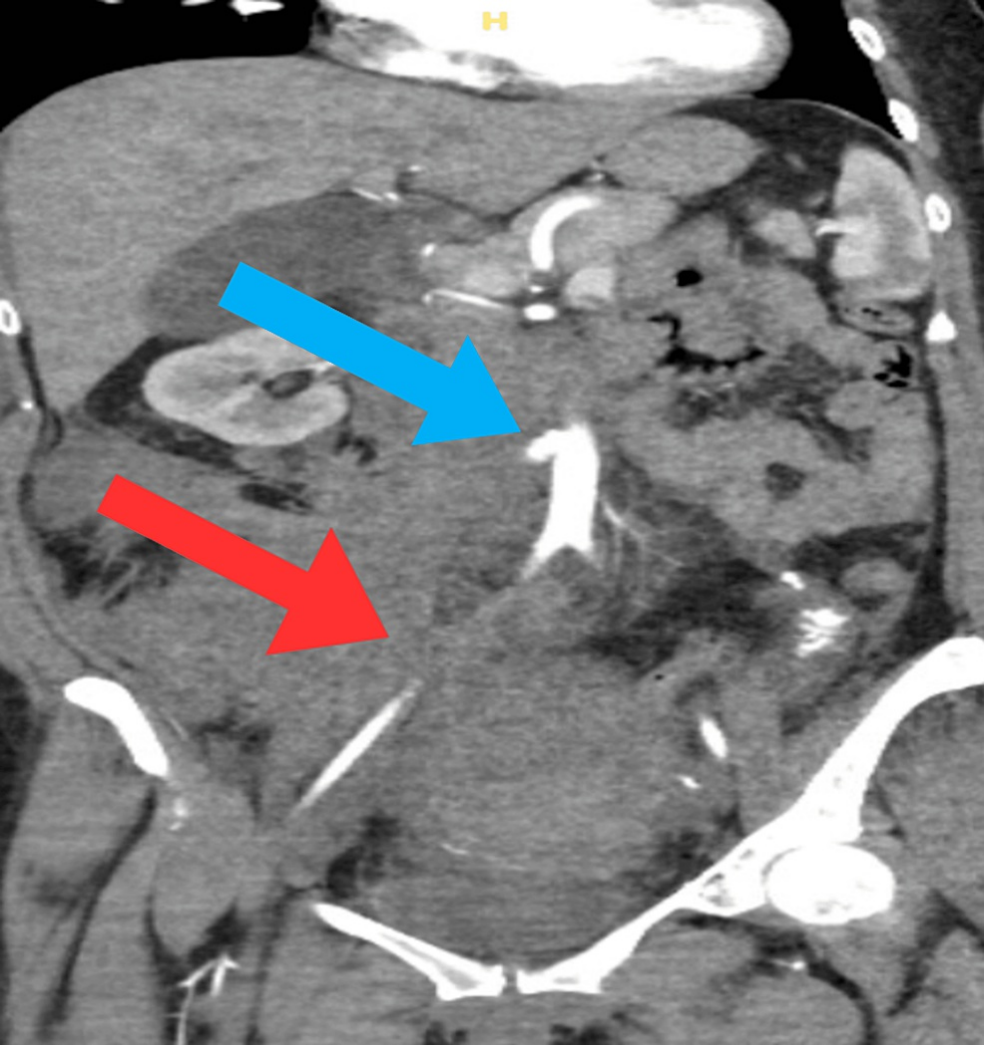
Right retroperitoneal hematoma (red arrow). Right dilated ovarian artery (blue arrow).

A 34-year-old G6P5015 female gave birth to a healthy baby boy via spontaneous vaginal delivery. The placenta was expelled intact, and the patient remained stable immediately after delivery, with an estimated blood loss of 110 mL. However, approximately 28 hours postpartum, a rapid response was initiated due to a significant drop in the patient's blood pressure to 58/33. The patient presented as pale, diaphoretic, and tachycardic, with a painful and visibility-distended abdomen. Treatment was initiated with Levophed (Pfizer Inc., New York, United States) (norepinephrine bitartrate), intravenous fluids, packed red blood cells (pRBC), cryoprecipitate, fresh frozen plasma (FFP), and platelets. Additionally, the patient underwent a CT angiogram of the abdomen and pelvis, revealing an ectatic right ovarian artery with areas of aneurysmal dilation and a large right retroperitoneal hematoma (Figure 1).

**Figure 2:**
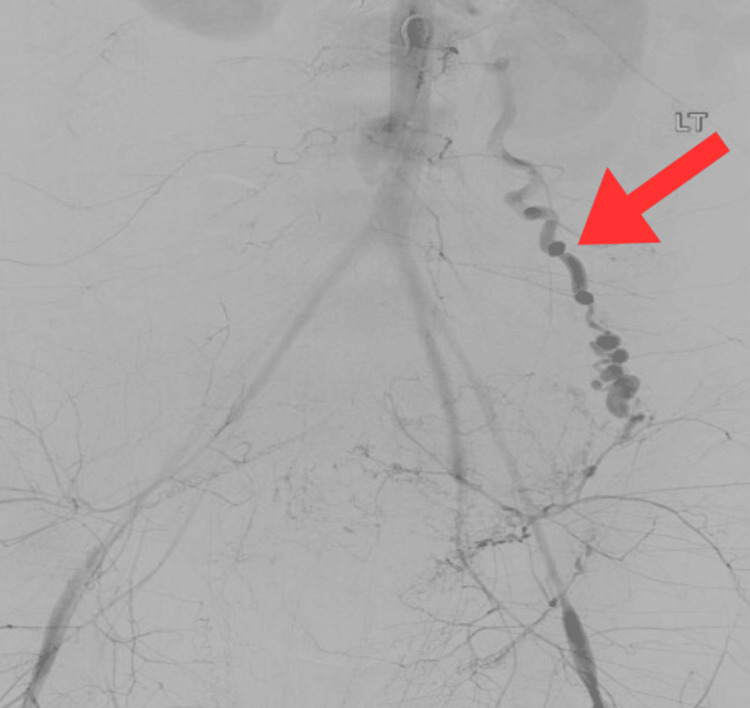
Left ovarian artery with distal saccular dilatations in a beaded appearance consistent with pseudoaneurysms.

The patient was taken to the operating room for an exploratory laparotomy (ex-lap) approximately 29 hours after giving birth. Upon entering the intraperitoneal cavity, a large amount of blood was noted, leading to poor visibility. The retroperitoneal space was also accessed, where a hematoma containing five liters of blood was evacuated. The ovarian arteries were ligated bilaterally with Weck clips. One liter of blood was evacuated from the intraperitoneal cavity for a total estimated blood loss of six liters. Despite the surgery, the patient remained hypotensive, and controlling the bleeding was challenging. As a result, interventional radiology (IR) was consulted for a more proximal embolization. A pelvic arteriogram and a TAE of the bilateral uterine and ovarian arteries were performed. We used 250 cc of Isovue (Bracco, Milan, Italy) (iopamidol) as the intravascular contrast, with the right common femoral artery serving as the access point. The arteriogram showed evidence of a torturous, hypertrophied left ovarian artery with distal saccular dilatations in a beaded appearance, consistent with pseudoaneurysms (Figure 2). After the emobilization, the patient was admitted to the intensive care unit (ICU), where she received vasopressors and transfusions while remaining intubated. Measurement of serial hemoglobin levels ordered during the rapid response revealed a nadir of 6.3 g/dL.

**Figure 3:**
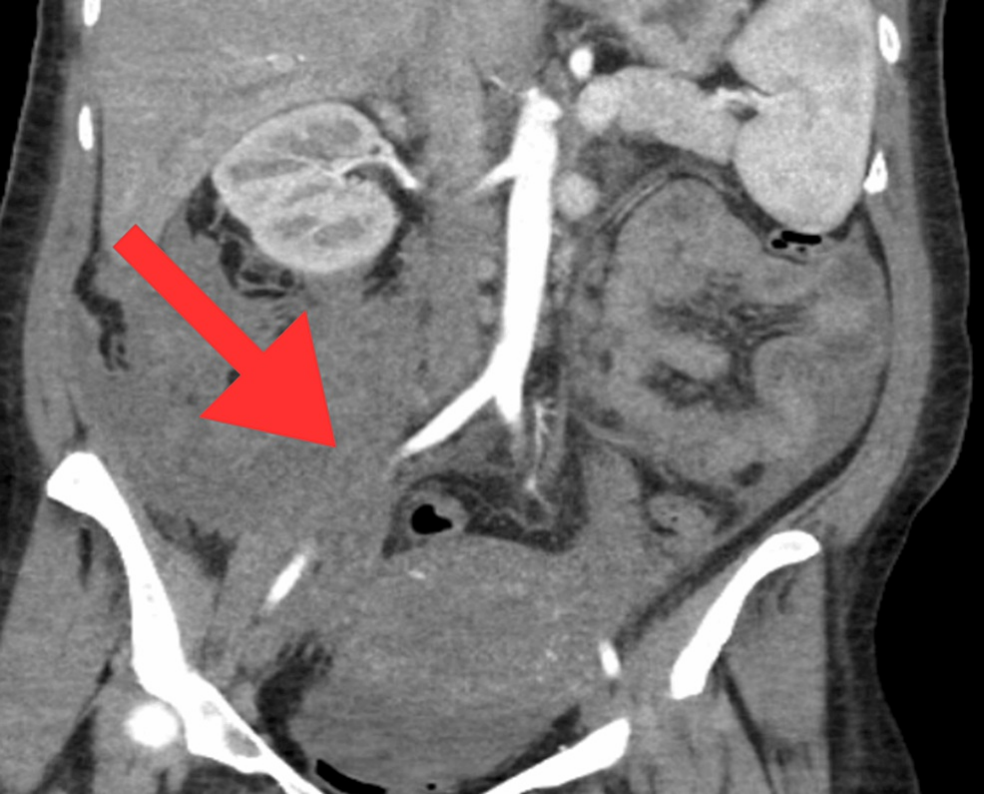
Resolving right retroperitoneal hematoma.

In the ICU, the patient was noted to be neurologically intact while intubated, with a Glasgow coma scale score of 10T. The patient was extubated and taken off vasopressors approximately 24 hours after the embolization, as blood pressure had stabilized and hemoglobin levels were trending between seven and eight grams per deciliter. In total, the patient received 17 units of pRBC, four units of cryoprecipitate, four units of FFP, and four units of platelets. Creatinine, aspartate transaminase (AST), and alanine aminotransferase (ALT) were within normal limits throughout the hospital course. Given the presence of abnormal vasculature, a vasculitis workup was ordered, including a rheumatoid factor, antinuclear antibodies (ANA), antineutrophil cytoplasmic antibodies (c-ANCA), perinuclear antineutrophil cytoplasmic antibodies (p-ANCA), myeloperoxidase (MPO) antibodies, anti-Jo-1 antibody, anti-Smith antibody, anti-ribonucleoprotein (anti-RNP) antibody, anti-double-stranded DNA (anti-dsDNA) antibody, anti-Ro antibody, and anti-La antibody, which all came back negative. The patient was downgraded to the postpartum floor about 48 hours after the embolization. Complications during the hospital course included bilateral pleural effusions, likely due to the extensive transfusions, which were managed with Lasix (Sanofi India Limited, Mumbai, India) (furosemide). Additionally, the patient developed thromboses in the left brachial, basilic, and cephalic veins, likely a consequence of immobility. Eliquis (Pfizer Inc., New York, United States) (apixaban) was administered under close hematological supervision. The patient also experienced significant pain with respirations that improved through her hospital course. This was likely due to the unabsorbed blood in the peritoneal cavity. Throughout her hospital stay, the patient was cared for by a multidisciplinary team that included cardiology, hematology, and intensive care. She was discharged 12 days post-delivery in stable condition and had a hemoglobin of 10.6 g/dL. A repeat CT angiogram of the abdomen and pelvis confirmed a resolving right retroperitoneal hematoma (Figure 3).

## Discussion

Retroperitoneal hemorrhage can be a potentially life-threatening complication resulting from a variety of diseases, including vascular, neoplastic, or coagulopathic disorders. It can even be a complication of procedures involving endovascular access, such as cardiac catheterization [8]. Presentation typically involves signs of severe hypotension from bleeding and sudden-onset flank or back pain. A rare pathology that may cause retroperitoneal hemorrhage is the rupture of an aneurysm of the ovarian artery. Ovarian artery aneurysm ruptures are typically associated with pregnancy in the puerperal period, although there are documented cases unrelated to pregnancy. Most reported cases are unilateral and occur in multiparous women. There are approximately 31 documented cases in the literature [9].

While the etiology of the formation of these aneurysms is not exactly known, most theories are centered on the hormonal changes that occur during pregnancy and the effect they have on the vasculature. Pregnancy-induced hemodynamic changes, such as increased blood volume and cardiac output may be involved. Other pregnancy-related changes related to the rapid growth of the uterus and its involution postpartum may also be involved in arterial changes that predispose to aneurysm formation [2,4].

Due to the acute nature of this pathology, rapid identification and diagnosis are imperative to successful outcomes. Hemodynamic support with isotonic crystalloids and potentially blood products may be immediately necessary. CT angiography of the abdomen and pelvis can be used to identify the aneurysm and potential extravasation from the aneurysm site into the retroperitoneal space [3].

Several of the literature accounts of OAA rupture were stabilized with TAE only, while others involved exploratory laparotomy and ovarian artery ligation followed by TAE, as in this case. Whether initial treatment with TAE or exploratory laparotomy is most appropriate may vary depending on the presentation of the patient and their hemodynamic stability [10]. In the case of this patient, a decision to proceed with exploratory laparotomy was made due to the acute nature of her presentation, but if time had permitted, TAE with evacuation of the retroperitoneal hematoma could have likely offered sufficient treatment and perhaps spared the patient an open surgery. To the best of our knowledge, only one other case has been reported that eventually revealed bilateral ovarian artery aneurysms, or pseudoaneurysms. In both cases, only one side ruptured, and the other side was discovered on imaging; however, both cases involved ligation of the unruptured aneurysm in addition to the ruptured one [11].

Patients with significant blood loss may also require continued hemodynamic support post-operatively with peripheral vasopressors and other ICU-level management. Close monitoring may reveal persistently low hemoglobin or requirements for other blood transfusion products. Although retroperitoneal hemorrhage due to any cause is a potentially life-threatening emergency, if recognized early, it can have successful outcomes. One retrospective study revealed that among 78 cases of retroperitoneal hematoma due to any cause, only two patients died due to hemorrhagic shock, and 59% were able to be managed medically alone without surgical intervention. However, this study also identified a significant risk factor for mortality, which is surgical intervention more than six hours after the initial injury, emphasizing the importance of rapid identification [12]. 

Despite this low mortality rate, retroperitoneal hematoma, specifically due to spontaneous hemorrhage as opposed to trauma, may be more complex to manage. Another retrospective study that evaluated spontaneous retroperitoneal hemorrhage revealed that 75% of patients had such significant blood loss that transfusion was required [13]. Due to its rarity and lower clinical suspicion when not involving blunt trauma, providers may not include it in their differential assessment. However, spontaneous hemorrhage into the retroperitoneum may occur, and providers should have raised suspicion for rupture of an OAA when assessing patients in the puerperal period, especially those with multiparity.

## Conclusions

A spontaneous rupture of an ovarian artery aneurysm, or pseudoaneurysm, is an incredibly rare event in the postpartum period. Still, it should remain in the differential in the event of a postpartum hypotensive episode, especially with multiparous women. More research is needed to develop a treatment algorithm for this condition. However, a transcatheter arterial embolization with or without an exploratory laparotomy is an effective option for managing these cases.
